# Enhanced expression of FCER1G predicts positive prognosis in multiple myeloma

**DOI:** 10.7150/jca.37313

**Published:** 2020-01-01

**Authors:** Lin Fu, Zhiheng Cheng, Fen Dong, Liang Quan, Longzhen Cui, Yan Liu, Tiansheng Zeng, Wenhui Huang, Jinghong Chen, Ying Pang, Xu Ye, Guangsheng Wu, Tingting Qian, Yang Chen, Chaozeng Si

**Affiliations:** 1 Department of Hematology, The Second Affiliated Hospital of Guangzhou Medical University, Guangzhou, 510260, China.; 2 Translational Medicine Center, State Key Laboratory of Respiratory Disease, The Second Affiliated Hospital of Guangzhou Medical University, Guangzhou 510260, China.; 3 Department of Hematology, Huaihe Hospital of Henan University, Kaifeng, China.; 4 Department of Pathology and Medical Biology, University Medical Center Groningen, University of Groningen, Groningen, Netherlands.; 5 Institute of Clinical Medical Sciences, China-Japan Friendship Hospital, Beijing, 100029, China.; 6 Translational Medicine Center, Huaihe Hospital of Henan University, Kaifeng, China.; 7 Department of Biomedical Sciences, University of Sassari, Sassari, 07100, Italy.; 8 Department of Hematology, First Affiliated Hospital, Medical College of Shihezi University, Shihezi 832008, China.; 9 MOE Key Laboratory of Bioinformatics; Bioinformatics Division and Center for Synthetic & Systems Biology, TNLIST; Department of Automation, Tsinghua University, Beijing, 100084, China.; 10 Department of Operations and Information Management, China-Japan Friendship Hospital, Beijing, 100029, China.

**Keywords:** Multiple myeloma, FCER1G, Prognosis, Gene expression profile, Bioinformatics analysis

## Abstract

**Background:** Multiple myeloma (MM) is the second most common hematologic malignancy worldwide and does not have sufficient prognostic indicators. *FCER1G* (Fc fragment Of IgE receptor Ig) is located on chromosome 1q23.3 and is involved in the innate immunity. Early studies have shown that *FCER1G* participates in many immune-related pathways encompassing multiple cell types. Meanwhile, it is associated with many malignancies. However, the relationship between MM and *FCER1G* has not been studied.

**Methods:** In this study, we integrated nine independent gene expression omnibus (GEO) datasets and analyzed the associations of *FCER1G* expression and myeloma progression, ISS stage, 1q21 amplification and survival in 2296 myeloma patients and 48 healthy donors.

**Results:** The expression of *FCER1G* showed a decreasing trend with the advance of myeloma. As ISS stage and 1q21 amplification level increased, the expression of *FCER1G* decreased (P = 0.0012 and 0.0036, respectively). MM patients with high *FCER1G* expression consistently had longer EFS and OS across three large sample datasets (EFS: P = 0.0057, 0.0049, OS: P = 0.0014, 0.00065, 0.0019 and 0.0029, respectively). Meanwhile, univariate and multivariate analysis indicated that high *FCER1G* expression was an independent favorable prognostic factor for EFS and OS in MM patients (EFS: P = 0.006, 0.027, OS: P =0.002,0.025, respectively).

**Conclusions:** The expression level of *FCER1G* negatively correlated with myeloma progression, and high *FCER1G* expression may be applied as a favorable biomarker in MM patients.

## Background

Multiple myeloma (MM) is a hematologic malignancy characterized by the monoclonal expansion of bone marrow plasma cells (BMPCs) 1-3. The International Staging System (ISS) divides MM into three categories based on the levels of β2-microglobulin and albumin at diagnosis, which are surrogate markers of tumor burden. Additionally, 1q21 amplification is considered a high-risk genetic feature, which is the most common chromosomal aberration in MM 4, 5. In recent years, genetic biomarkers are starting to play an increasingly important role in the prognosis of myeloma 6, 7. Therefore, it is necessary to investigate novel biomarkers to predict the prognosis of MM, so as to help improve the prognostication and treatment of MM.

*FCER1G* is a protein coding gene located on chromosome 1q23.3 8. It has been reported that *FCER1G* interacts with other factors and participates in various nuclear pathways 9. Specifically, *FCER1G* is a constitutive component of the high-affinity immunoglobulin E (IgE) receptor and interleukin-3 receptor complex. It is mainly involved in mediating the allergic inflammatory signaling of mast cells, selectively mediating the production of interleukin 4 (IL4) by basophils, and initiating the transfer from T-cells to the effector T-helper 2 subset 10, 11. It also forms a functional signaling complex together with the pattern recognition receptors *CLEC4D* and *CLEC4E* in myeloid cells. Previous studies have shown that *FCER1G* is an innate immunity gene and may be involved in the development of eczema, meningioma and childhood leukemia 12-14. *FCER1G* is associated with the progression of clear cell renal cell carcinoma (ccRCC) and may improve prognosis by affecting the immune-related pathways. In addition, *FCER1G* is underexpressed in acute myeloid leukemia 15. Moreover, *FCER1G* is a critical molecule in signaling pathways that are widely involved in a variety of immune responses and cell types 16. However, the prognostic role of *FCER1G* in MM remains largely unknown.

Here, we explored the relationship between* FCER1G* expression and myeloma progression, ISS stage, 1q21 amplification, and survival, using the gene expression data of 2296 MM patients and 48 healthy donors. We were able to demonstrate that high expression of *FCER1G* was a good indicator of MM and was related to positive outcomes.

## Methods

### Data source

In this study, we selected 2296 myeloma patients and 48 healthy donors from the Gene Expression Omnibus database (GEO). In order to assess the relationship between *FCER1G* expression and the prognosis of MM patients, the sample was divided into two cohorts. In the first cohort, there were six independent microarray datasets (GSE39754, GSE5900, GSE2113, GSE6477, GSE47552, GSE13591). This cohort included 48 healthy donors and 640 MM patients in different stages of monoclonal gammopathy (104 monoclonal gammopathy of undetermined significance (MGUS), 69 smoldering myeloma (SMM), 452 multiple myeloma (MM) and 15 plasma cell leukaemia (PCL)). This cohort was used for microarray expression analysis.

The second cohort consisted of three big independent microarray datasets of MM patients, GSE2658, GSE4204 and GSE24080. In GSE2658, the gene expression data of 559 MM patients was evaluated by the Affymetrix Human Genome U133 Plus 2.0 Array. Samples in GSE4204 were pre-treatment bone marrow aspirates from 538 MM patients. In GSE24080, the gene expression profiling of highly purified bone marrow plasma cells was performed in 559 newly diagnosed MM patients. This cohort was mainly used for survival analysis, and the expression of *FCER1G* in different 1q21 amplification levels and different ISS stages was also described.

All the samples were classified according to the International Myeloma Working Group criteria 17. The diagnosis of MM (ICD-10 C90.0) was established in accordance with the World Health Organization guidelines^18^. The diagnosis of MGUS require more than 10% plasma cell infiltration in the bone marrow, while the levels of monoclonal protein could not exceed 30 g/L and there would be no evidence of related organ or tissue impairment (ROTI) defined as hypercalcemia, renal impairment, anemia, or bone lesions attributed to plasma-cell proliferation. SMM was defined with bone marrow plasmacytosis exceeding 10%, monoclonal protein level greater than 30 g/L, in the absence of ROTI 19. The diagnostic definition of PCL is based on Kyle's criteria, where peripheral blood plasma cell absolute count greater than 2 × 10^9^/L or percentage of the while blood cells more than 20% 20, 21.

In GSE39754, the DNA microarray data of CD138+ myeloma cells from 170 newly diagnosed MM patients, and plasma cells (PCs) from 6 normal donors, were quality controlled and normalized with the aroma Affymetrix package. The gene expression level was estimated with a probe level model (PLM) 22. In GSE5900, International Myeloma Working Group criteria were used to classify patients as having MGUS, SMM, or symptomatic MM 19. In GSE6477, Bone marrow aspirate samples were obtained and enriched for CD138+ cells. In GSE64552, bone marrow samples were obtained from 20 patients with MGUS, 33 with high-risk SMM and 41 with MM. All samples corresponded to newly diagnosed untreated patients 22. In GSE2113, the gene expression profiles of purified plasma cells (PCs) were purified from bone marrow Series, after red blood cell lysis with 0.86% ammonium chloride, using CD138 immunomagnetic microbeads 22. In GSE13591, pathological bone marrow specimens from 41 MM and 4 plasma cell leukemia (PCL) patients at diagnosis (27 males; median age 67 years, range 46-85) were obtained. The plasma cells of the samples were purified (≥90%) from the bone marrow samples. Samples in GSE2658 and GSE4204 were pre-treatment bone marrow aspirates from multiple myeloma patients 23, 24. The GSE24080 dataset was contributed by the Myeloma Institute for Research and Therapy at the University of Arkansas for Medical Sciences (UAMS, Little Rock, AR, USA). Gene expression profiling of highly purified bone marrow plasma cells was performed in newly diagnosed patients with MM. Plasma cells were enriched by anti-CD138 immunomagnetic bead selection of mononuclear cell fractions of bone marrow aspirates in a central laboratory 25.

All clinical and molecular information and microarray datasets of these patients were publicly accessible at the Gene Expression Omnibus (http://www.ncbi.nlm.nih.gov/geo). All experiment design, quality control, and data normalization were in line with the standard Affymetrix protocols. The research was conducted in accordance with the International Conference and the Declaration of Helsinki.

### Microarray analysis

All microarray data were identified in GEO, and we employed statistical analysis to investigate significantly abnormally expressed genes on every microarray dataset. Briefly, gene expression data were obtained by using Affymetrix human Genome 133 plus 2.0. All designs and quality control of the microarray experiment and data normalization were in line with the standard Affymetrix protocols. Patients with *FCER1G* expression values above the median for all MM patients were classified as *FCER1G*^high^, and the others were considered to be *FCER1G*^low^. *P*-value < 0.05 in unpaired t-test analysis and fold change (FC, log2) > 0.5 or < -0.5 was utilized to determine the differential expression of genes (DEGs).

### Statistical analysis

All statistical analysis was performed by R software 3.5.0. Each dataset was first evaluated for normality of distribution by the Kolmogorov-Smirnov test to decide whether a non-parametric rank-based analysis or a parametric analysis should be used. The Fisher exact and Wilcoxon rank-sum tests were used to test hypotheses in categorical and continuous variables, respectively. The samples in the second cohort were divided into two groups (*FCER1G*^high^, n = 280, *FCER1G*^low^, n = 279) based on the median expression values of FCER1G. Different gene expression analysis was performed by the limma package 26. The Kaplan-Meier method and Cox regression multivariate analysis were used to estimate the survival analysis, with group comparisons made by using the log-rank test. Clusterprofiler package was used to identify GO enrichment terms and KEGG pathways 27. For all statistical analysis, *P*-value< 0.05 was considered significant.

## Results

### The expression level of *FCER1G* decreased with the progression of multiple myeloma

**Figure 1:**
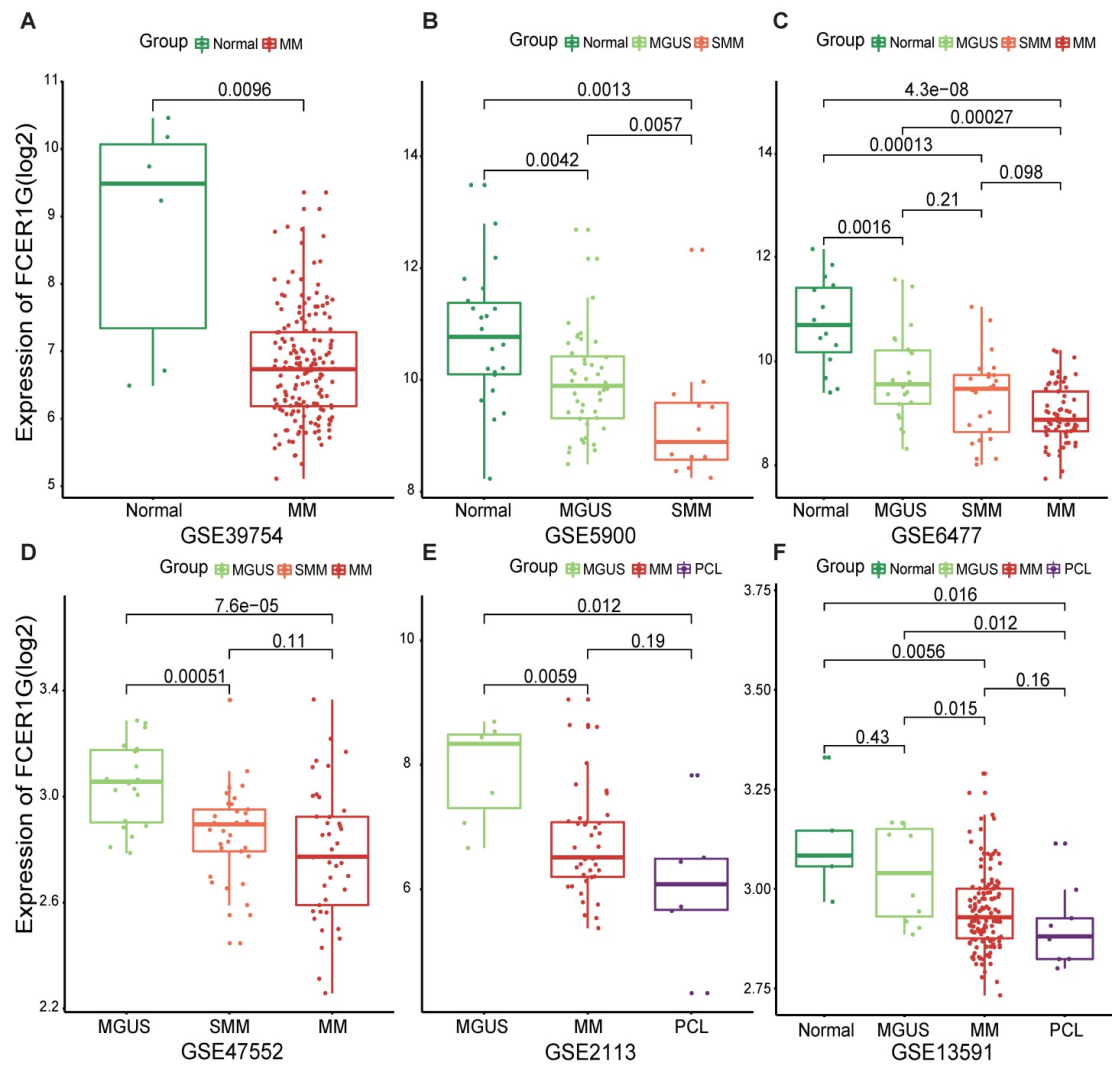
** The expression level of *FCER1G* in several GEO datasets of Normal and myeloma patients in different stages.** The X-axis represents the sample type, the Y-axis represent *FCER1G* expression level(log2). **A** MM patients (n= 170) compared with Normal samples (n= 6). **B** The different expression of *FCER1G* in Normal (n= 22), MGUS (n= 44), and SMM (n= 12). **C** Expression value of *FCER1G* in Normal (n= 15) and other different stages of 115 myeloma patients. MGUS (n= 22), SMM (n= 24), and MM (n= 69).** D** *FCER1G* expression level in different subtypes of myeloma patients. MGUS (n= 20), SMM (n= 33), MM (n= 41).** E** Comparison of *FCER1G* expression levels in 3 different stages of myeloma patients: MGUS (n= 7), MM (n= 39), and PCL (n= 6). **F** The correlation of *FCER1G* expression level between Normal (n= 5) and 3 different myeloma stages: MGUS (n= 11), MM (n= 133), and PCL (n= 9).

In order to understand the expression of *FCER1G* in MM patients and other different myeloma stages, we employed six datasets to analyze the expression level of it. We observed that the expression level of *FCER1G* decreased with the progression of myeloma. Remarkably lower expression of *FCER1G* was found in 170 MM patients than in 6 normal donors (P = 0.0096, Fig. 1A). In GSE5900, a significant decrease of *FCER1G* expression in Normal (n = 22), MGUS (n = 44) and SMM (n = 12) was noticed (P = 0.0042, 0.0057, 0.0013, severally, Fig. 1B). An obvious downtrend of *FCER1G* expression alongside the progression of disease was further validated in GSE6477, including Normal (n = 15), MGUS (n = 22), SMM (n = 24) and MM (n = 69) (P = 0.0016, 0.21, 0.096, 0.00013, 0.00027, 4.3e-08, respectively, Fig. 1C). Moreover, the expression level of *FCER1G* decreased from MGUS (n = 20) to SMM (n = 33) and MM (n = 41), (P = 0.00051, 0.11, 7.6e-05 severally, Fig. 1D). The same trend was also found in GSE2113 dataset among MGUS (n = 7), MM (n = 39), and PCL (n = 6) (P = 0.0059, 0.19, 0.012, Fig. 1E), as well as in the GSE13591 dataset including normal donor (n = 5), MGUS (n = 11), MM (n = 133) and PCL (n = 9) (Fig. 1F). In summary, the expression of *FCER1G* decreased with the evolution of monoclonal gammopathy, suggesting that *FCER1G* might be involved in the malignant progression of myeloma.

### The expression of *FCER1G* in MM patients between different ISS stages

**Figure 2:**
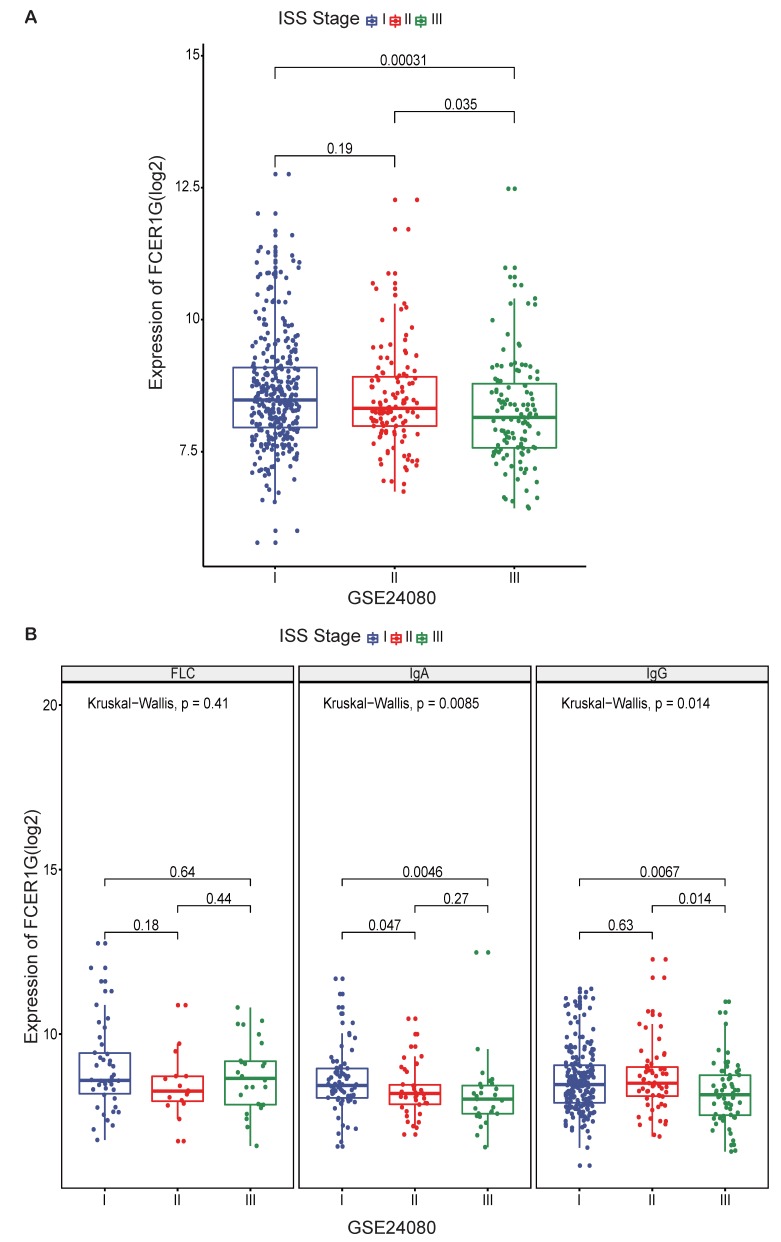
** The expression of *FCER1G* in different ISS stages of MM patients.** The X-axis represents the ISS stage while the Y-axis represents *FCER1G* expression value(log2). **A** The expression level of *FCER1G* had a decreasing trend with the ISS stage increases, Kruskal-Wallis test. **B** *FCER1G* expression pattern in different serotypes. FLC, *P*= 0.41, IgA, *P*= 0.0085, IgG, *P*= 0.014 respectively, Kruskal-Wallis test.

To further investigate the value of *FCER1G* expression, we compared the expression level of *FCER1G* at different ISS stages in 559 MM patients. A trend of decreasing *FCER1G* expression level in stages I, II and III (Fig. 2A, P = 0.19, 0.035, 0.00031). We also compared the expression of *FCER1G* in different serotypes of different ISS stages. In serum immunoglobulin A (IgA) group and serum immunoglobulin G (IgG) group, the expression of *FCER1G* in stage I, II and III decreased gradually. However, there was no statistical significance in the serum free light chain (FLC) group (Fig. 2B, FLC: P = 0.41, IgA: P = 0.0085, IgG: P = 0.014, Kruskal-Wallis test). These results indicated that low expression of *FCER1G* correlated with the severity of MM.

### Differences in clinical and other classic prognostic biomarkers in MM between *FCER1G*^high^ and *FCER1G*^low^ groups

Using the GSE24080 dataset of 559 MM patients, we also analyzed the baseline characteristics between high and low *FCER1G* expression groups. We divided the samples into two groups based on the median value of *FCER1G* expression: *FCER1G*^low^ (n = 279) and *FCER1G*^high^ (n = 280). Between the two groups, there are no significant differences in the demographic factors, such as age, gender and race. However, *FCER1G* was more likely to be associated with isotype (P = 0.019), cytogenetic abnormality (P = 0.021) and different therapy options (P = 0.013). Additionally, the MM patients with low *FCER1G* expression were more likely to have a higher beta-2 microglobulin (B2M), creatinine (CREAT), aspirate plasma cells (ASPC) ,bone marrow biopsy plasma cells (BMPC) and lower hemoglobin (HGB), which were all important factors in MM prognosis (*P<* 0.001, = 0.038, < 0.001, < 0.001, = 0.006, respectively). Moreover, the *FCER1G*^low^ group was more likely to have a higher expression of *CDK4*, *GPRC5D*, *HK2*, *TP53* (P = 0.002, 0.001, 0.005, 0.008, respectively) and lower expression of *WT1*, *CXCL12, DEK*, *CD74*, *DAPK3*, *FGFR3*, *XBP1*, *KISS1*, *IGHG1*, *MS4A1*, *RGS13*, *S1PR1* (P = 0.006, <0.001, <0.001, =0.001, =0.004, <0.001, <0.001, =0.01, <0.001, <0.001, <0.001, <0.001) (Table 1).

### Prognostic value of *FCER1G* expression in MM

By using the Cox regression model, we computed multivariate hazard ratios for different variables of 559 MM patients. Univariate analysis results showed that *FCER1G* and albumin (ALB), beta-2 microglobulin (B2M), bone marrow biopsy plasma cells (BMPC), hemoglobin (HGB), number of magnetic resonance imaging (MRI) were all closely related to EFS and OS with significant P values (Table 2). Furthermore, in multivariate analysis for EFS, the hazard ratio of hemoglobin was 0.66 (P = 0.023), while the hazard ratio of *FCER1G* expression was 0.7 (P = 0.024). These two factors were significantly related to the EFS in MM patients. For OS, the hazard ratio of beta-2 microglobulin was 1.66 (P = 0.007) and the hazard ratio of *FCER1G* expression was 0.69 (P = 0.02), indicating that both had a close association with OS. The hazard ratio of albumin and number of magnetic resonance imaging were 0.58 and 1.93 (P = 0.001, <0.001). *FCER1G* expression value was a stable factor affecting the survival level of MM patients (Table 3).

### The expression of *FCER1G* in different amplification levels of 1q21

**Figure 3:**
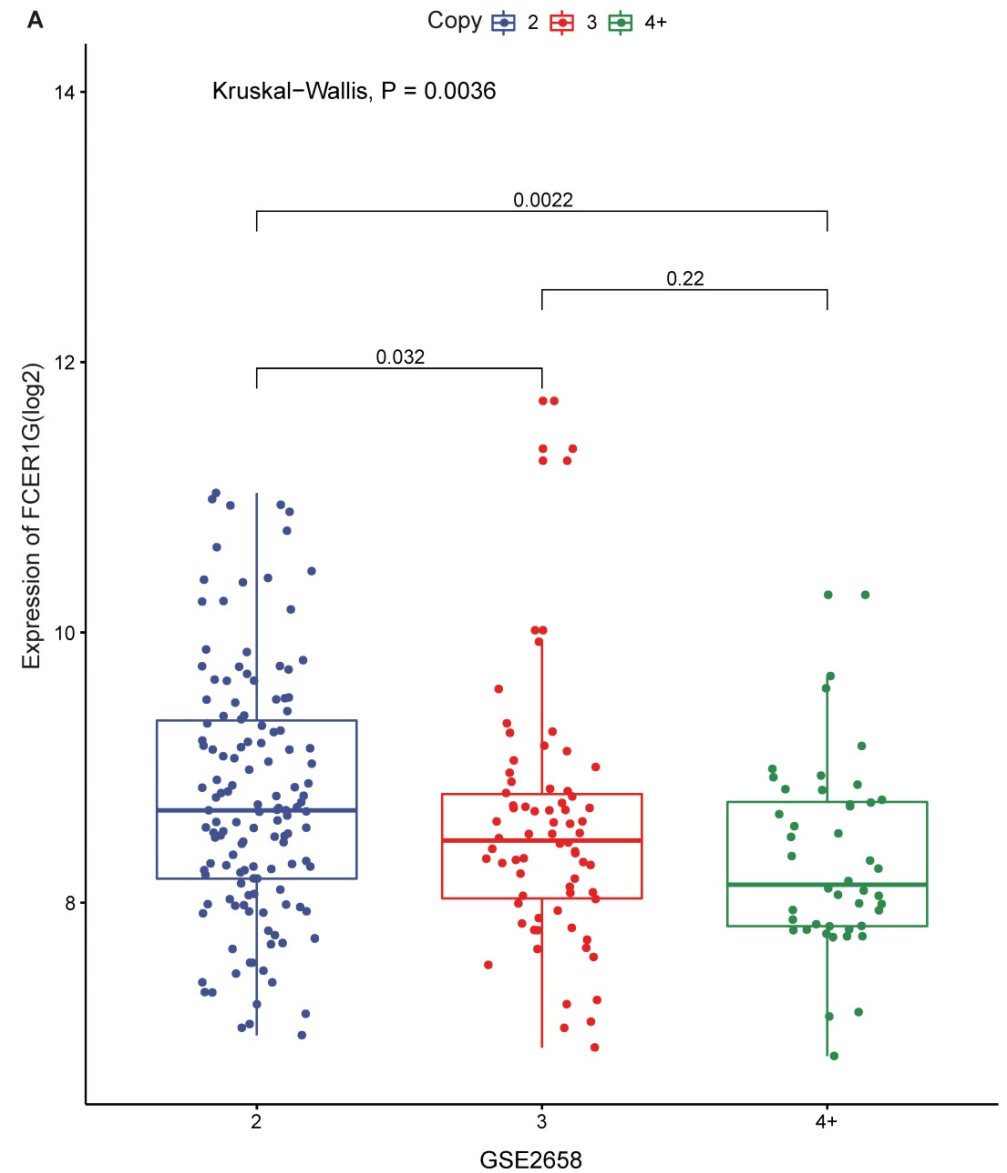
** The expression of *FCER1G* in different amplification levels of 1q21.** The X-axis represents the 1q21 amplification, the Y-axis represents the *FCER1G* expression level. **A** *FCER1G* expression levels at different amplification levels of 1q21 in 248 MM patients. The expression value was measured as log2. *P*= 0.022, Kruskal-Wallis test.

1q21 amplification is associated with poor prognosis, and *FCER1G* is located on chromosome 1q23.3. We compared *FCER1G* expression level under the different amplification of 1q21. There was a statistically significant difference of the expression levels between different levels of 1q21 amplification. The expression of *FCER1G* was decreased with the amplification of 1q21 (Fig. 3A, P = 0.0036, Kruskal-Wallis test).

### *FCER1G* predicts the survival level in MM

**Figure 4:**
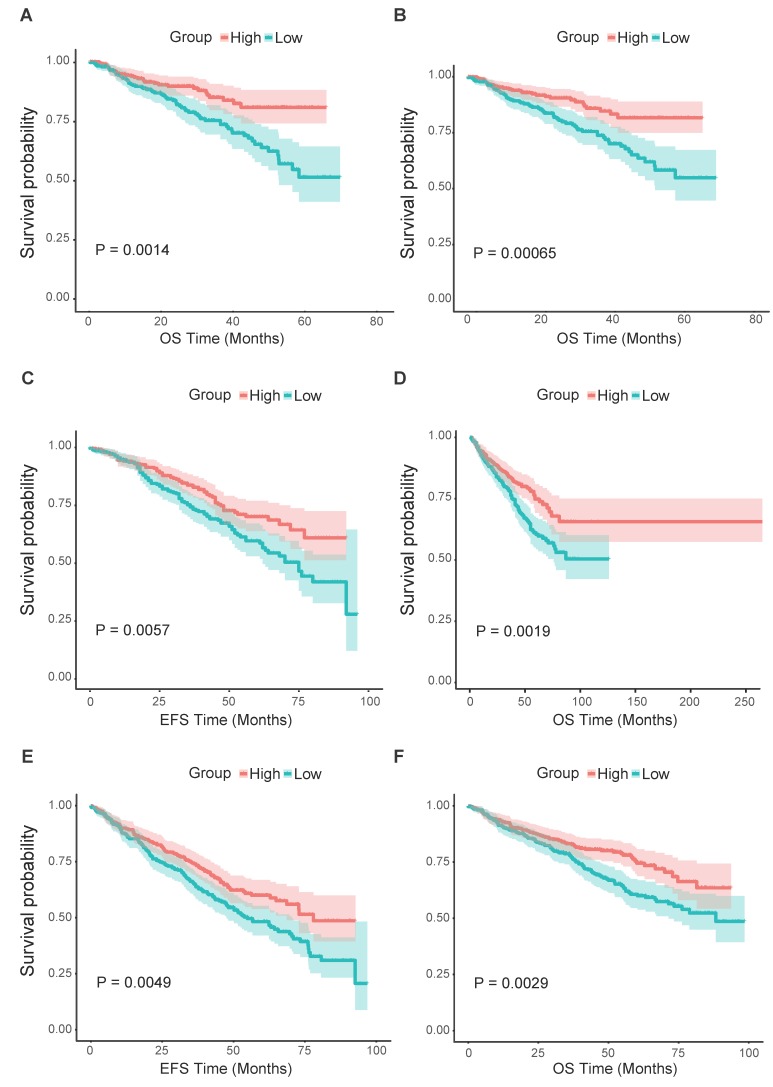
** Survival analysis of *FCER1G*^high^ and *FCER1G*^low^ group.** The X-axis represents the survival time(month) and the Y-axis represents survival probability. Kaplan-Meier survival curves showed that *FCER1G*^high^ predicts good endpoint in both event-free survival time (EFS) and overall survival (OS), Log-rank test. **A** OS between *FCER1G*^high^ and *FCER1G*^low^ in GSE2658 dataset with *P*= 0.0014. **B** OS analysis in GSE2404 of 538 pre-treatment MM patients with *P*= 0.00065. **C, D** The survival analysis of EFS and OS in *FCER1G*^high^ and *FCER1G*^low^ groups of 559 MM patients in GSE24080 dataset. EFS: *P*= 0.0057, OS: *P*= 0.0019. **E, F** The EFS and OS results at the milestone points of 2008 in GSE24080 with *P*= 0.0049, *P*= 0.0029 respectively.

From all the results above, we could assume that the low expression of *FCER1G* was related to adverse outcomes of MM. Thus, we further analyzed the survival level in the second cohort. We found that the *FCER1G*^low^ group had significantly shorter OS compared to the *FCER1G*^high^ group in two independent datasets of GSE2658 and GSE4204 (Fig. 4A and B, P = 0.0014, 0.00065, respectively). The same prognostic value of *FCER1G* in MM was also found in GSE24080 (Fig. 4C and D, OS, P = 0.0019, EFS, P = 0.0057). Likewise, the survival level retains similar results at the milestone points of Year 2008 (Fig. 4E and F, OS, P = 0.0029, EFS, P = 0.0049).

### Different expression and pathway analysis for DEGs of *FCER1G*^high^ versus *FCER1G*^low^

**Figure 5:**
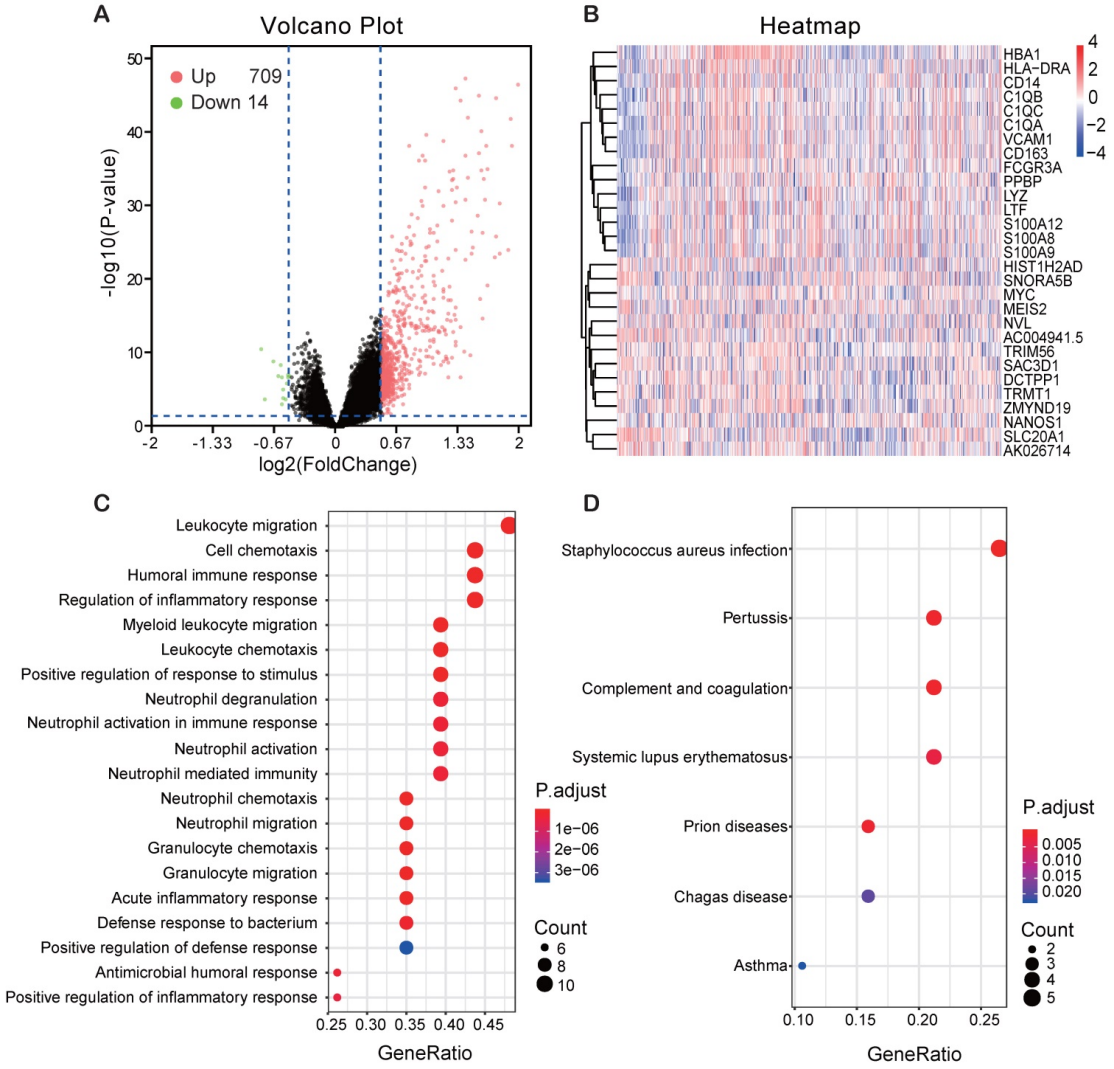
** Different expression genes (DEGs) and the results of GO enrichment and KEGG pathway analysis. A** Volcano plot of the DEGs expression between *FCER1G*^high^ and *FCER1G*^low^. Cut-off criteria for DEGs significance was *P*< 0.05 and the absolute value of the log2 fold change> 0.5. The Y-axis displays the -log10 *P*-value for each gene, while the X-axis displays the log2 fold change for that gene relative to *FCER1G* expression. Green dots represent 14 down-regulated genes, the red circle represents 709 up-regulated genes, and black dots indicate non-significance genes. **B** Heatmap shows top 15 up-regulated genes and top 14 down-regulated genes. The red represents high expression, the white represents intermediate expression, and the blue represents low expression. **C, D** GO and KEGG results for differential expression genes. The X-axis represents gene ratio and the Y-axis represents different enriched pathways.

In order to find the genes associating with *FCER1G*, we analyzed the differential expression values of *FCER1G*^high^ versus *FCER1G*^low^. As many as 709 genes were up-regulated and 14 genes were down-regulated (*P*< 0.05, (FC, log2)> 0.5 or < -0.5, Fig. 5A). Heatmap showed the top 15 up-regulated genes and 14 down-regulated genes (Fig. 5B). By using the DEGs, we analyzed the enriched GO terms and KEGG pathways. Among the biological process terms of GO, most of DEGs were enriched in leukocyte migration (GO:0050900), cell chemotaxis (GO:0060326), humoral immune response (GO:0006959), and regulation of inflammatory response (GO:0050727) (Fig. 5C). In the KEGG analysis results, Staphylococcus aureus infection (hsa05150), Systemic lupus erythematosus (hsa05322) and complement and coagulation cascades (hsa04610) were the most enriched pathways (Fig. 5D).

### Module screening from the PPI network

**Figure 6:**
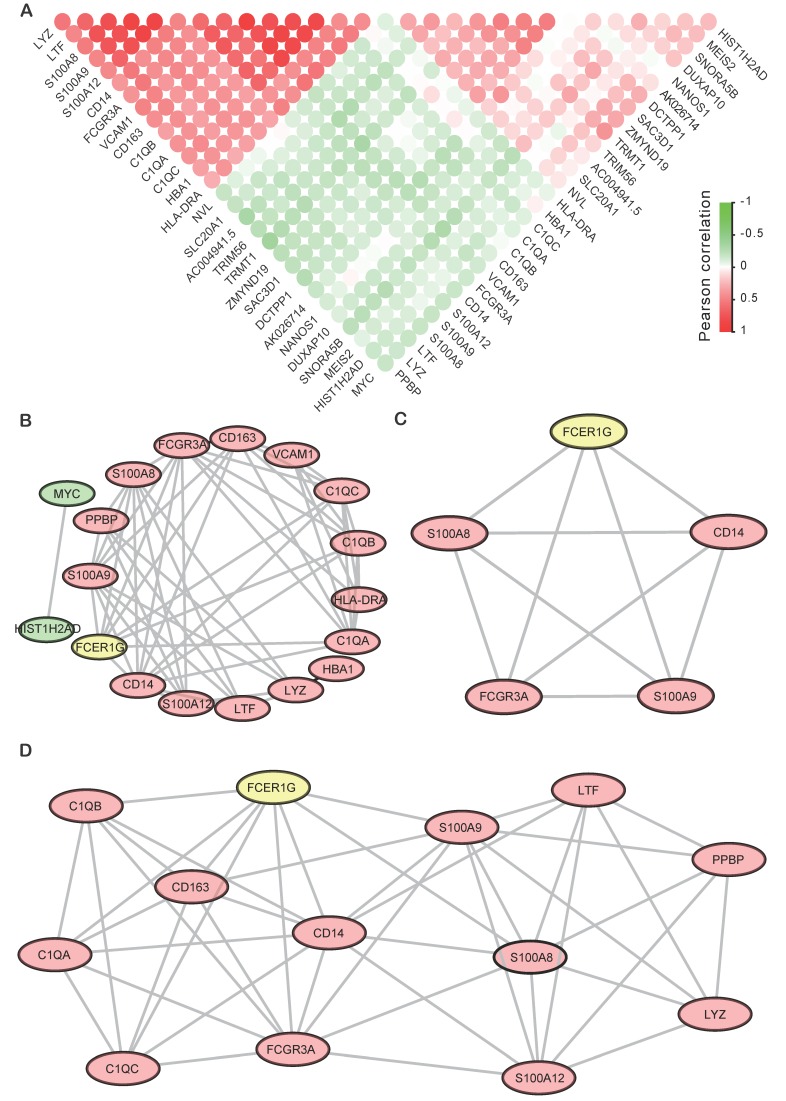
** The Correlation analysis and PPI results of DEGs. A** The correlation analysis of DEGs with the Pearson correlation coefficient, the red circle means positive correlation while green means negative correlation. **B** PPI network of top DEGs of top 15 up-regulated genes and 14 down-regulated genes. **C, D** Sub-networks analysis of the PPI network by using MCODE APP in Cytoscape.

Finally, all the top 29 DEGs of *FCER1G*^high^ versus *FCER1G*^low^ were used to calculate the correlativity between those genes (Fig. 6A). We also screened the protein-protein interaction (PPI) network in the String database by using the top 29 DEGs 28. Most of the up-regulated genes and two down-regulated genes (*MYC* and *HIST1H2AD*) were interactional in the PPI network (Fig. 6B). Then we discovered two sub-networks by using MCODE in Cytoscape (Fig. 6C, D). In the PPI network, *C1QB, C1QA, C1QC, CD163, CD14, S100A8, S100A9, LTF, LYZ* and *FCGR3A* were all reported to be associated with MM in early research. *FCER1G* acts as a core gene in both the general network and two subnetworks.

## Discussion

Our research demonstrated that the expression level of *FCER1G* showed a decreasing trend in the deterioration of plasma cell malignancy. Higher expression of *FCER1G* in MM patients was associated with favorable prognosis. Likewise, the GO and KEGG pathways mainly enriched in defense response, immune response, and inflammatory response. PPI network also revealed that many cancer-associated genes interacted with *FCER1G*. All of these results show that *FCER1G* may be a tumor suppressor gene in myeloma.

Early studies found that *FCER1G* transduced activation signals from various immunoreceptors 10, 29. It was functionally linked to mediate neutrophil activation and was also involved in platelet activation. Associated diseases included Bleeding Disorder, Platelet-Type, 11(BDPLT11) and Mitochondrial Complex I Deficiency. *FCER1G* also engaged in many immune responses and played a tumor-promoting role in many kinds of tumors, such as meningioma, Clear cell renal cell carcinoma (ccRCC), childhood leukemia and Acute Myeloid Leukemia(AML) 12, 14, 15, 30. It was also reported that the demethylation of *FCER1G* was induced by IL15 in the NKp30+CD8+ T cell population exhibiting high natural killer-like antitumor potential 31. *FCER1G* inhibits the expression of certain Alzheimer's disease susceptibility genes by participating in Herpes simplex (HSV-1) escape strategy 9. Interestingly, the abundant expression of *FCER1G* was found in the circulating tumor cells of a prostate cancer patient who was sensitive to docetaxel chemotherapeutic reagent 32.

In the PPI network, many important genes that were associated with MM had been screened. *S100A9* was reported significantly down-regulated in MM patients and further support MM survival by stimulating angiogenesis and cytokine secretion 33, 34. *S100A9* was directly implicated in promoting Myeloid-derived suppressor cells (MDSC), which plays a critical role in the MM progression and can be considered as a therapeutic target in this disease 35. *C1QB*, *C1QA*, and *C1QC* were all the complement c1q chains. Early reports showed that complement c1q acts in the tumor micro environment as a cancer-promoting factor independently of complement activation 36. *LTF* has identified as a Cereblon (CRBN) binding protein and established relevance to MM biology 37. *LYZ* was found as an element of the 9-genes prognostic signature and might be an independent prognostic factor in patients with multiple myeloma 38. *HLA-DRA* plays an important role in bone lesions common in MM patients by participating in the immune response activation pathway 39. *CD163* is a tumor-associated macrophage marker, the high level of Monocyte/macrophage-derived soluble *CD163* was associated with higher stage according to the ISS and with other known prognostic factors in multiple myeloma 40, 41. *MYC* activation is associated with hyperdiploid MM and shorter survival, and also plays a causal role in the progression of monoclonal gammopathy to multiple myeloma. *MYC* protein overexpression is a feature of progression and adverse prognosis in multiple myeloma 42-45. In recent research, it was also proved that sialyltransferase inhibition leads to inhibition of tumor cell interactions with *VCAM1*, and improves survival in a human multiple myeloma mouse model 46. *FCGR3A* was proved to be associated with anti-tumor response. 47. The polymorphisms of *FCGR3A* play an important role in First-Relapsed ovarian cancer, metastatic breast cancer, and metastatic colorectal cancer 48-51. Early research also found that *FCGR3A* was associated with infections of MM patients 52.

The PPI results showed that *FCER1G* was a hub gene in the network and directly interact with many MM associated genes. As we demonstrated previously, downregulation of *FCER1G* expression was closely related to the deterioration of myeloma. Combined with GO and KEGG analysis results above, *FCER1G* might interact with other MM associated genes and was mainly involved in leukocyte migration, cell chemotaxis, and immune and inflammatory response pathway, and therefore exerted an anti-cancer effect in multiple myeloma.

## Conclusions

To sum up, we have clearly demonstrated that high *FCER1G* expression was a good prognostic factor in MM patients. The expression level of *FCER1G* decreased with the progression of myeloma. Moreover, GO term enrichment, KEGG pathways, and PPI networks involved in MM provided insights into the pathogenesis processes associated with varying *FCER1G* expression. The underexpression of *FCER1G* could serve as a promising therapeutic target for MM patients.

However, in this research, the exact pathophysiologic role of *FCER1G* in myeloma cells was not been fully demonstrated. Further studies including the molecular mechanism and deeper genomic research of *FCER1G* in myeloma deterioration will be urgently required.

## Figures and Tables

**Table 1:** Patients' characteristics in the GSE24080 dataset of 559 MM patients according to FCER1G expression levels.

		FCER1G^low^, n=279	FCER1G^high^, n=280	*P*-value
AGE, mean(range)		57.39(29.70-76.50)	56.97(24.83-75)	0.787
Gender (%)	female	116(41.58)	106(37.86)	0.417
	male	163(58.42)	174(62.14)	
RACE (%)	other	27(9.68)	35(12.5)	0.353
	white	252(90.32)	245(87.5%)	
ISOTYPE (%)	FLC	35(12.54)	49(17.50)	0.019
	IgA	79(28.32)	54(19.29)	
	IgG	148(53.05)	165(58.93)	
B2M (mean(sd))		5.625(6.62)	3.842(3.53)	<0.001
CRP (mean(sd))		11.986(24.25)	11.278(21.72)	0.717
CREAT (mean(sd))		1.434(1.46)	1.211(1.05)	0.038
LDH (mean(sd))		173.05(69.06)	170.907(62.77)	0.701
ALB (mean(sd))		4.033(0.58)	4.065(0.59)	0.511
HGB (mean(sd))		11.042(1.84)	11.464(1.76)	0.006
ASPC (mean(sd))		46.779(23.63)	38.431(24.36)	<0.001
BMPC (mean(sd))		52.49(25.53)	40.262(25.63)	<0.001
MRI (mean(sd))		11.197(14.65)	10.872(14.44)	0.799
Cytogenetic abnormality (%)	No	162(58.06)	190(67.86)	0.021
	Yes	117(41.94)	90(32.14)	
High CCND1, no (%)		130(46.59)	150(53.57)	0.118
High WT1, no (%)		123(44.09)	157(56.07)	0.006
High CXCL12, no (%)		71(25.45)	209(74.64)	<0.001
High DEK, no (%)		107(38.35)	173(61.79)	<0.001
High CD74, no (%)		119(42.65)	161(57.5)	0.001
High NRAS, no (%)		143(51.25)	137(48.93)	0.642
High CDK4, no (%)		159(56.99)	121(43.21)	0.002
High BRAF, no (%)		150(53.76)	130(46.43)	0.099
High LIG4, no (%)		134(48.03)	146(52.14)	0.374
High GPRC5D, no (%)		160(57.35)	120(42.86)	0.001
High DAPK3, no (%)		122(43.73)	158(56.43)	0.004
High FGFR3, no (%)		105(37.63)	175(62.5)	<0.001
High XBP1, no (%)		116(41.58)	164(58.57)	<0.001
High KISS1, no (%)		124(44.44)	156(55.71)	0.01
High PTPN11, no (%)		139(49.82)	141(50.36)	0.966
High IDH2, no (%)		144(51.61)	136(48.57)	0.526
High HRAS, no (%)		139(49.82)	141(50.36)	0.966
High HK2, no (%)		157(56.27)	123(43.93)	0.005
High IGHG1, no (%)		109(39.07)	171(61.07)	<0.001
High MS4A1, no (%)		110(39.43)	170(60.71)	<0.001
High RGS13, no (%)		115(41.22)	165(58.93)	<0.001
High RRAS2, no (%)		131(46.95)	149(53.21)	0.163
High S1PR1, no (%)		112(40.14)	168(60)	<0.001
High TP53, no (%)		156(55.91)	124(44.29)	0.008
Therapy (%)	TT2	187(67.03)	158(56.43)	0.013
	TT3	92(32.97)	122(43.57)	

AGE: Age at registration (years); B2M: Beta-2 microglobulin, mg/l; CRP: C-reactive protein, mg/l; CREAT: Creatinine, mg/dl; LDH: Lactate dehydrogenase, U/l; ALB: Albumin, 35 g/l; HGB: Haemoglobin, g/dl; ASPC: Aspirate plasma cells (%); BMPC: Bone marrow biopsy plasma cells (%); MRI: Number of magnetic resonance imaging (MRI)- defined focal lesions (skull, spine, pelvis); Cytogenetic abnormality: An indicator of the detection of cytogenetic abnormalities; no: number of patients.

**Table 2:** Univariate analysis for EFS and OS.

Variables	EFS		OS	
	HR(95%CI)	P-value	HR(95%CI)	P-value
FCER1G(high vs. low)	0.65(0.48-0.88)	0.006	0.62(0.45-0.84)	0.002
AGE(≥60 vs. <60)	0.97(0.71-1.32)	0.839	1.4(1.04-1.89)	0.028
Gender	1.05(0.77-1.43)	0.75	0.97(0.72-1.32)	0.85
ALB	0.76(0.56-1.02)	0.071	0.49(0.36-0.67)	< 0.001
B2M	1.72(1.27-2.33)	< 0.001	2.21(1.64-3)	< 0.001
BMPC	1.63(1.18-2.27)	0.003	1.82(1.29-2.56)	0.001
HGB	0.54(0.39-0.74)	< 0.001	0.62(0.45-0.84)	0.002
MRI	1.26(0.93-1.71)	0.141	1.9(1.38-2.61)	< 0.001

EFS: event-free survival; OS: overall survival; CR: complete remission; HR: hazard ratio; CI: confidence interval; ALB: Albumin(35 g/l); B2M: Beta-2 microglobulin(mg/l); BMPC: Bone marrow biopsy plasma cells (%); HGB: Haemoglobin(g/dl); MRI: Number of magnetic resonance imaging (MRI)- defined focal lesions (skull, spine, pelvis).

**Table 3:** Multivariate analysis for EFS and OS

Variables	EFS		OS	
	HR(95%CI)	P-value	HR(95%CI)	P-value
FCER1G(high vs. low)	0.7(0.51-0.95)	0.024	0.69(0.51-0.94)	0.02
AGE(≥60 vs. <60)	0.91(0.66-1.24)	0.549	1.3(0.96-1.76)	0.086
Gender	1.13(0.82-1.54)	0.451	0.99(0.73-1.34)	0.949
ALB	0.85(0.62-1.16)	0.313	0.58(0.42-0.79)	0.001
B2M	1.26(0.88-1.82)	0.209	1.66(1.15-2.39)	0.007
BMPC	1.31(0.91-1.88)	0.145	1.34(0.92-1.96)	0.13
HGB	0.66(0.46-0.94)	0.023	0.95(0.67-1.34)	0.756
MRI	1.3(0.96-1.77)	0.095	1.93(1.4-2.65)	< 0.001

EFS: event-free survival; OS: overall survival; CR: complete remission; HR: hazard ratio; CI: confidence interval; ALB: Albumin, 35 g/l; B2M: Beta-2 microglobulin, mg/l; BMPC: Bone marrow biopsy plasma cells (%); HGB: Haemoglobin, g/dl; MRI: Number of magnetic resonance imaging (MRI)- defined focal lesions (skull, spine, pelvis).

## Abbreviations

MM: Multiple myeloma
FCER1G: Fc fragment Of IgE receptor Ig
BMPCs: bone marrow plasma cells
ISS: International Staging System
IgE: immunoglobulin E
DEGs: different expression genes
MGUS: monoclonal gammopathy of undetermined significance
MM: multiple myeloma
SMM: smouldering myeloma
PCL: plasma cell leukaemia
GEO: gene expression omnibus
PCs: purified plasma cells
ROTI: related organ or tissue impairment
B2M: beta-2 microglobulin
CREAT: Creatinine
ASPC: Aspirate plasma cells
HGB: hemoglobin
MRI: magnetic resonance imaging
OS: overall survival
EFS: event-free survival
HR: hazard ratio
CI: confidence interval
GO: gene Ontology
KEGG: Kyoto encyclopedia of genes and genomes
PPI: Protein-protein interaction
BP: biological processes
CC: cell component
MF: molecular function
MDSC: Myeloid-derived suppressor cells
